# Event-specific detection of transgenic potato AV43-6-G7 using real-time and digital PCR methods

**DOI:** 10.1186/s12896-016-0303-8

**Published:** 2016-10-27

**Authors:** Hongwei Gao, Xiaofan Yu, Tingting Deng, Min Sun, Xizhi Xiao, Xin Huang, Ying Chen, Ronggui Li

**Affiliations:** 1 Shandong Entry-Exit Inspection and Quarantine Bureau of People’s Republic of China, Qingdao, China; 2 Qingdao University Medical College, Qingdao, China; 3 Chinese Academy of Inspection and Quarantine Institute, Beijing, China

**Keywords:** Transgenic, Potato AV43-6-G7, Flanking sequence, Event Specific, Real-time PCR, Detection

## Abstract

**Background:**

The isolation of unknown DNA sequences flanked by known sequences is an important task in the event-specific detection of GMOs. None of event-specific detection method was developed based on the junction sequence of an exogenous integrant in the transgenic potato AV43-6-G7.

**Results:**

The flanking sequence between the exogenous fragment and recombinant chromosome of this potato was successfully acquired through exogenous gene 5′-RACE. The event-specific primers and *Taq*man probe were designed to amplify fragments spanning the exogenous DNA and potato genomic DNA. The specific real-time PCR and digital PCR detection methods for AV43-6-G7 potato were established based on primers designed according to the flanking sequences. The detection limit of the qualitative PCR assay was 0.01 % for AV43-6-G7 potato in 100 ng of potato genomic DNA, corresponding to approximately 11.6 copies of the potato haploid genome. The ddPCR assays for Potato AV43-6-G7 achieved a limit of quantification of approximately 58 target copies, with RSD ≤ 25 %. The aLOQ of this system was approximately 1.2 copies.

**Conclusions:**

These results indicated that these event-specific methods would be useful for the identification of potato AV43-6-G7.

**Electronic supplementary material:**

The online version of this article (doi:10.1186/s12896-016-0303-8) contains supplementary material, which is available to authorized users.

## Background

Since the first genetically modified tomato was approved, the number and production of genetically modified (GM) crops have continuously increased [1]. According to the International Service for the Acquisition of Agri-Biotech Applications (ISAAA), 29 types of plants and 390 transgenic events were developed and commercialized worldwide by the end of March 2016 [2]. However, the perceived risks of genetically modified organisms (GMOs) to food safety and the environment have raised concerns [3]. To provide consumers with information on food, many countries have developed legislations to determine the threshold of GMO ingredients. The threshold for mixing GMOs in feed and foodstuffs are 0.9 % in EU, 3 % in Korea, 5 % in Japan, and 1 % in New Zealand and Australia [4]. In China, the mandatory labelling of GMO products is required without setting a clear threshold value according to the Regulations on Administration of Agricultural GMOs Safety [5].

The isolation of unknown DNA sequences flanked by known sequences is an important task in the event-specific detection of GMOs. Both rapid amplification of cDNA ends (RACE) and thermal asymmetric interlaced PCR (TAIL-PCR) are effective methods for this purpose [6, 7]. Fraiturea et al. [8] also used a DNA walking strategy using a genome walking kit on rice products. To comply with GMO labelling regulations and ensure product legality and traceability, event-specific detection methods are essential for GMO identification and quantification. Event-specific detection was primarily performed at the DNA level using polymerase chain reaction (PCR)-based methods because of its high specificity for the flanking sequence of the exogenous integrant. In case of quantitative analysis, the Taqman-based real-time quantitative PCR (qPCR) method is preferred, reflecting the high accuracy (how close a measured value is to the actual value) and precision (how close the measured values are to each other) of this method. However, this method has some obvious drawbacks. Notably, PCR inhibitors, such as chloroform, ionic detergents, etc., the experience of the technicians, and the matrix effect of the PCR tubes affect standard curve-based qPCR [9]. Particularly, the significant bias observed when the target is present at low concentrations could seriously limit the use of qPCR [9] Another requirement for qPCR is reference material for GMO at serial concentrations, as standard curves for quantities of endogene and exogene are prepared separately. The lack of certified reference material has been also a hindrance for qPCR. Despite the shortcomings of qPCR, this method is convenient for qualitative detection and cheap in terms of instruments and reagents compared with dPCR.

Digital PCR (dPCR) has rapidly developed since it was first conceived in the 1992 [10]. In dPCR, the reaction mixture is distributed in a large number of partitions containing zero, one or more copies of the target nucleic acid. After end-point amplification, the signal would be positive (1) or negative (0) for partitioned samples [10]. The absolute number of target nucleic acid molecules in the original sample prior to partitioning can be calculated from the ratio of positive to total partitions using Poisson distribution . Compared with the real-time PCR methods, PCR inhibitors do not impact dPCR, and this method does not need reference material for GMO at serial concentrations [11]. Digital PCR also is an effective approach to detect SNP variants in a diluted target sample [12, 13]. The development and application of absolute quantitative detection through the duplex chamber-based digital PCR of genetically modified maize events has been reported [13]. Stevanato demonstrated that dPCR could differentiate all varieties of sugar beet samples carrying specific SNP alleles associated with flowering at low concentrations (1 %) of the target sequence, while qPCR and HRM showed only a moderate frequency (10 %) [12].

Thus, this technique is better suited for international-trade applications. Currently, two designated dPCR technologies are commercially available: micro fluidic chamber digital PCR (cdPCR) and emulsion droplet digital PCR (ddPCR). Both commercial systems enable suitable GMO testing. According to our experience, each sample would cost approximately US $12.1 in ddPCR, while each sample should cost US $150 to US $400 per chip or plate in cdPCR [14]. In addition, ddPCR has higher repeatability than cdPCR according to the results of preliminary experiments using three genes [data not shown].

Potato is one of the first important transgenic plants [15]. The main purposes of gene transfer technology for potato were to improve the quality of potatoes and enhance pest-resistant varieties. Monsanto, a company in US, developed a range of anti-coleopteran pests and transgenic potatoes against leafroll virus prior to 1997 [16]. Recently, the quality of potato starch has been improved in more commercial transgenic potatoes, such as transgenic potato Amflora (EH92-527-1 potato) and transgenic potato AM04-1020 [17, 18]. These transgenic potatoes produce amylose-free starch through the antisense inhibition of the gene encoding granule bound starch synthase I (GBSSI) [19]. These improved functionalities are not only promising for technical applications in paper and textile, but also in the food industry for the production of dairy products, soups, sauces and noodles. The transgenic potato AV43-6-G7 in the present study is a variety of starch potato intended for starch isolation by the starch industry, and the starch might undergo further processing.

In the present study, the exogenous gene fragment and flanking sequence of transgenic potato AV43-6-G7 were obtained using the 5′-RACE method. According to information about the determined sequence, a pair of primers and a Taqman probe were designed based on the exogenous fragment and endogenous chromosomal sequence of potatoes. An event-specific detection method was developed for the transgenic potato AV43-6-G7 using real-time PCR and ddPCR.

## Methods

### Text materials and DNA preparation

A list of all test materials is provided in Additional file 1 the Supporting Information. All non-powder samples, including cottonseed, rapeseed, corn liquor dregs, and non-transgenic potatoes, were ground into a 60-mesh powder. The genomic DNA was extracted and purified using the Nucleic Acid Extraction System np968 (Tianlong Science and Technology Co., Ltd, Xi’an, China) in accordance with the manufacturer’s instructions. This DNA extraction method involves four successive steps: (1) each ground sample (50 mg) was added to 300 μL DNA extraction buffer; (2) the samples were homogenized and incubated with lysis buffer; (3) the samples were centrifuged, and the supernatant collected; and (4) the nucleic acids were extracted using magnetic beads.

The relative sensitivity of the samples was determined according to the procedure below. A series of different percentages 100 %, 10 %, 1 %, 0.1 %, 0.01 %, and 0.001 % (v/v) of the transgenic potato preps were made individually after mixing transgenic potato DNA-containing supernatant with non-transgenic potato DNA-containing supernatant. The 100 % transgenic potato preps were obtained after mixing 1000 μL of transgenic potato DNA-containing supernatant with 0 μL of non-transgenic potato DNA-containing supernatant, and 10 % transgenic potato preps were was obtained after mixing 100 μL of transgenic potato DNA-containing supernatant with 900 μL of non-transgenic potato DNA-containing supernatant, etc. The transgenic potatoes were converted into non-transgenic potatoes at 100 %, 10 %, 1 %, 0.1 %, 0.01 %, and 0.001 % (v/v). The quantity of DNA samples was measured at 260 nm and prepared into solutions of 10 ng/μL for real-time PCR and ddPCR. The DNA purity was evaluated based on A260/A280 ratios using a UV spectrophotometer (BioPhotometer plus, Eppendorf, Germany).

### Determination of the 5′-flanking DNA sequence

The 5′-flanking DNA sequence of transgenic potato AV43-6-G7 was determined using the 5′-RACE method. Based on the partial sequence of the lac gene in heterologous DNA in potato AV43-6-G7 [19], the 5′ ends were obtained through the rapid amplification of cDNA ends (RACE) using gene-specific and adapter primers. For 5′-RACE, PCR was performed using the gene-specific outer primer MAV43 R1 and primer AP1. The DNA sample was amplified a 25-μL reaction in a 0.2-mL tube containing 200 nM of primer gene-specific outer primer MAV43 R1 and primer AP1, 2× GC Buffer (TaKaRa DRR02AG, China), 2 mM magnesium chloride, 200 nM each of dATP, dCTP, dGTP, and dTTP, 1 unit of LA Taq (TaKaRa DRR02AG, China) and 100 ng of the extracted DNA template. The PCR program was set at 95 °C for 5 min, followed by 10 cycles at 94 °C for 30 s, 68 °C (less than 0.8 °C per cycle) for 30 s, and 72 °C for 150 s and 30 cycles at 94 °C for 30 s, 60 °C for 30 s and 72 °C for 150 s, with a final cycle at 72 °C for another 10 min.

Nested PCR was performed using the gene-specific outer primer MAV43 R2 and primer AP2. The DNA sample was amplified in a 25-μL reaction in a 0.2-mL tube containing 200 nM of each primer MAV43 R1 and primer AP1, 2× GC Buffer (TaKaRa DRR02AG, China), 2 mM magnesium chloride, 200 nM each of dATP, dCTP, dGTP and dTTP, 1 unit of LA Taq (TaKaRa DRR02AG, China), and100 ng extracted DNA template. The PCR was conducted at 95 °C for 5 min, followed by 28 cycles at 94 °C for 30 s, 58 °C for 30 s, and 72 °C for 150 s, with a final cycle at 72 °C for another 10 min.

### Oligonucleotide primers and probe

**Fig. 1:**
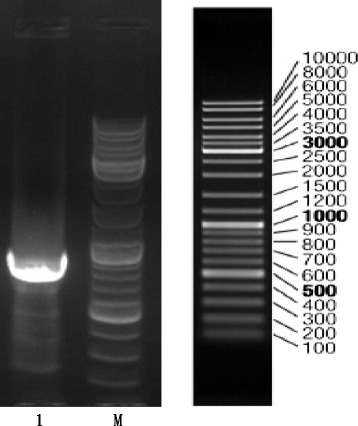
Electrophoresis of transgenic AV43-6-G7 potato in flanking region using the second PCR and 5′-RACE. 1: products of PCR using primers MAV43-R2 and AP2. M: DNA markers

The oligonucleotide primers and probe used in this study were designed using Primer Express version 3.0 (Applied Biosystems) and are listed in Table 1. For potato, the beta-fructosidase gene (fru) was selected as an endogenous reference gene [20]. The locations of the designed event-specific primer pairs and probes targeting the DNA sequence are shown in Fig. 1. All primers and probes were synthesized at Sangon Co. Ltd. (Shanghai, China).

**Table 1:** Primers and probe in this study

Name	Sequence and labled fluorescence (5′- -3′)	Origin
potato endogenous gene *fru* -F	CTGCCTCCGTCAAGATTTGGTCACT	[12]
potato endogenous gene *fru*-R	CTCTTCCCTTTCTTGATGG	[12]
potato endogenous gene *fru*-P	FAM-ACTTGTAATTCATCAAGCCAT- BHQ1	[12]
MAV43 R1	GCAAGCTTGGCGTAATCATGGTCATA	this study
MAV43-R 2	CGCTCACAATTCCACACAACATACGA	this study
AP1 primer	GTAATACGACTCACTATAGGGC	this study
AP 2 primer	ACTATAGGGCACGCGTGGT	this study
Potato AV43-6-G7 primerF	GGTATCAGGTTCTGGAATAAGACCAA	this study
Potato AV43-6-G7 primerR	TGTCGTGCCAGCTGCATTA	this study
Potato AV43-6-G7 Probe	FAM-CCCGCGCGTTGGCCGAT-BHQ1	this study
Potato AV43-6-G7 Probe used in duplex ddPCR	VIC-CCCGCGCGTTGGCCGAT-BHQ1	this study

### Qualitative PCR assays

The potato endogenous beta-fructosidase gene (fru) and event-specific sequence of GM potato AV43-6-G7 were separately amplified using potato AV43-6-G7 DNA and 24 negative non potato AV43-6-G7 DNA samples.

Real-time PCR was performed with a real-time PCR instrument (Master Cycle Realplex4, Eppendorf, Germany). Each DNA sample was amplified in a 25-μL reaction in a 0.2-mL tube containing 200 nM each of primer F, primer R, and probe, 10× Taq Buffer (Transgene, Beijing, China), 2 mM magnesium chloride, 200 nM each of dATP, dCTP, dGTP and dTTP, 1.5 units of Taq DNA polymerase (Transgen, Beijing, China), and 100 ng of extracted DNA template [21]. After initial denaturation at 95 °C for 120 s, the PCR conditions were optimized through 45 cycles of amplification (at 95 °C for 15 s, and 60 °C for 30 s). In each RT-PCR setup, the reaction systems were filled with purified water (non template control, NTC) and negative sample DNA as negative controls. The same baseline was used for the threshold cycle (Ct) to compare the RT-PCR performances of the extracted DNA and negative DNA samples. For sensitivity tests of event-specific primers and probes, the concentration of the DNA template ranged from 0.001 to 100 ng.

### Droplet digital PCR reactions and analysis

Droplet digital PCR was performed using a Bio-Rad QX100 droplet system (Bio-Rad, Pleasanton, CA, USA). The reaction mixtures contained 10 μL of 2× ddPCR™ Supermix (Bio-Rad, Pleasanton, CA, USA) for probe, 1 μL of primer and probe, and 5 μL of DNA template, in a total reaction volume of 20 μL. Each concentration was repeated in three parallel reaction cells. The optimized cycling program for ddPCR assays included a denaturation step at 95 °C for 10 min, followed by 45 cycles at 94 °C for30 s, 60 °C for 1 min, and 98°C for 10 min. After PCR cycling, the fluorescence signals were collected using a QX100 droplet reader. The data were acquired and analysed using Quanta Soft software [22].

The absolute sensitivity of the samples was realized after diluting 100 % genomic DNA to a 10 ng/μl solution. Subsequently, the 10 ng/μl solution was diluted 5 times at a 10-fold dilution.

Duplex droplet digital PCR reactions were performed using a FAM-labelled probe for the endogenous gene fru and a VIC-labelled probe for the AV43-6-G7-specific sequence at a concentration of 1 × 10^-5^ ng/μL [23], with 10 repeat reactions.

The detection data for ddPCR using Bio-Rad platforms was analysed using Bio-Rad analysis software (Bio-Rad). The number of positive partitions for both event-specific and endogenous amplifications for each sample were counted using Bio-Rad platforms. Based on the Poisson distribution, the original copy number of the different samples was calculated using the following equations [24]:

1 $$ \mathrm{A}\ \left(\mathrm{A}\mathrm{V}43\hbox{-} 6\hbox{-} \mathrm{G}7\ \mathrm{specific}\ \mathrm{sequence}\right)=\hbox{--} \ln\ \left[\left(\mathrm{N}\hbox{--} \mathrm{X}\right)/\mathrm{N}\right] \times \mathrm{N} $$

2 $$ \mathrm{B}\ \left(\mathrm{endogenous}\ \mathrm{gene}\ \mathrm{f}\mathrm{r}\mathrm{u}\right)=\hbox{--} \ln\ \left[\left(\mathrm{N}\hbox{--} \mathrm{Y}\right)/\mathrm{N}\right] \times \mathrm{N} $$

In the above two equations, A (AV43-6-G7 specific sequence) and B (endogenous gene fru) were the estimated copy numbers of the endogenous and event-specific genes for each panel. N was the total number of partitions; and X and Y were the positive wells for the AV43-6-G7 event-specific genes and the endogenous gene fru. The GMO proportion was calculated using the following equation:

3 $$ \frac{\mathrm{A}\left(\mathrm{A}\mathrm{V}43-6-\mathrm{G}7\ \mathrm{specific}\ \mathrm{sequence}\right)\ }{\mathrm{B}\left(\mathrm{endogenous}\ \mathrm{gene}\ \mathrm{f}\mathrm{r}\mathrm{u}\right)}\times 0.62 $$

In equation (3), 0.62 was used as a correction coefficient for the different copy numbers of endogenous gene and screening gene in the genome. Ten samples of pure GM potato AV43-6-G7 were utilized to examine the AV43-6-G7 event-specific sequence and the endogenous gene fru using digital PCR. The average ratio of AV43-6-G7 event-specific sequence and endogenous gene fru was 0.62.

## Results

### Characterization of the 5′-flanking region of transgenic AV43-6-G7 potato

According to information of “Overall Application of Amylopectin Potato Event AV43-6-G7 Based on Regulation (EC) on 1829/2003″ from the website (http://www.gmo-compass.org/pdf/regulation/potato/AV43-6-G7_application_food_feed_cultivation.pdf) [25], the integrated heterologous DNA in potato AV43-6-G7 is shown in Fig. 1. The DNA fragment (shown in Fig. 1) was derived from the 5′-flanking region using 5′-RACE.

**Fig. 2:**
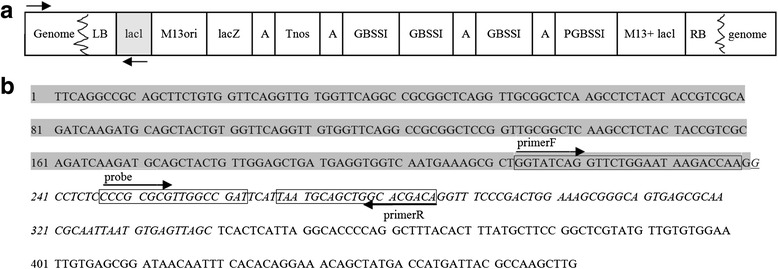
Frame of transgenic elements in the AV43-6-G7 potato genome. **a** Schematic diagram of the integrated exogenous gene in AV43-6-G7 potato, where ‘A’ means artificial poly linker sequence; **b** Flanking sequence of the potato genome and left border sequence of exogenous gene. The sequence in the grey background was obtained from the potato genome, while the other sequence was exogenous and obtained from the left border and the *lacl* gene. The sequence of primer F was obtained from the potato genome, and that of primer R and probe was obtained from the AV43-6-G7 potato

To identify and analyse the junction region of the exogenous integrant and the host genomic DNA of potatoes, 4 specific primers (MAV43 R1, MAV43-R 2, AP1, and AP2) were designed based on the sequence of the Lac operon from M13 mp 19 to amplify the 5′-flanking sequences using 5′-RACE. The RACE-PCR product was sequenced. Figure 2 shows the 5′-junction sequence of an exogenous integrant of the AV43-6-G7 potato. This sequence comprised a 239-bp fragment from M13 mp 19 and an unknown 221-bp DNA sequence.

**Fig. 3:**
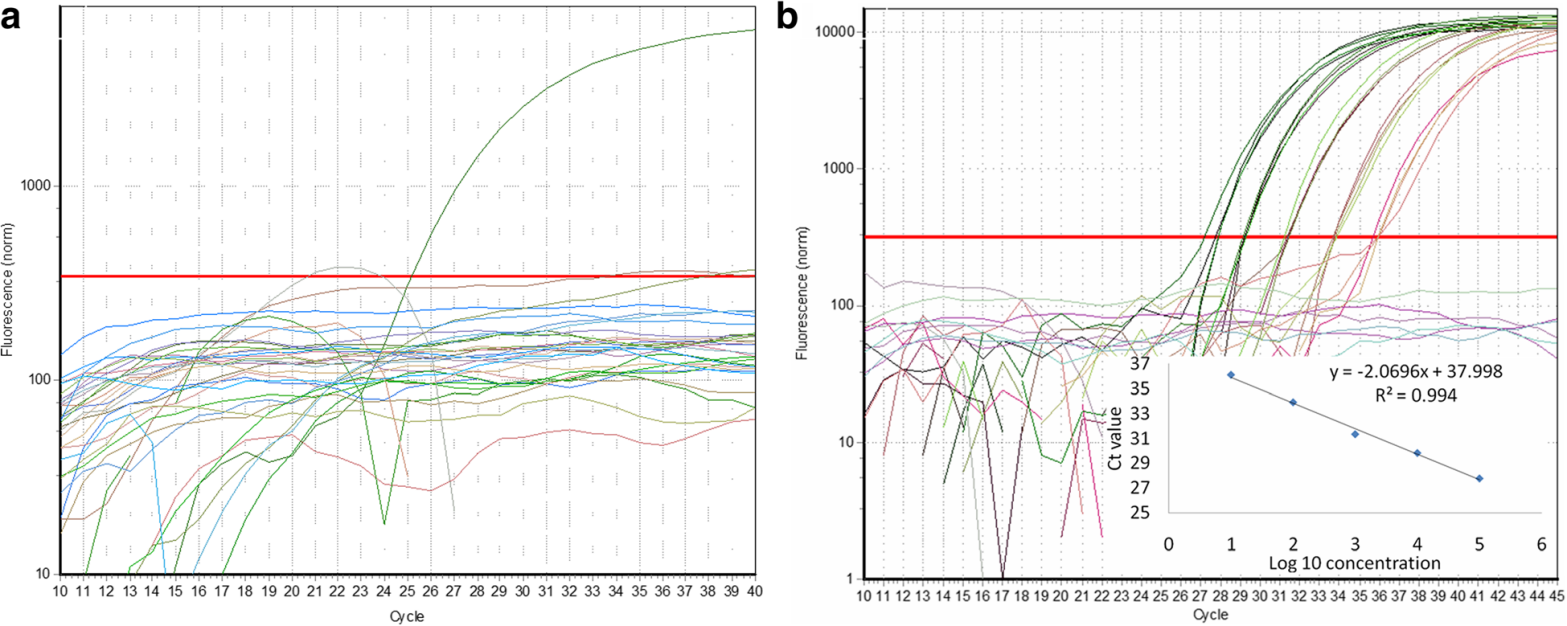
The amplification curves and standard curve for detecting GM AV43-6-G7 potato using real-time PCR. **a** Specificity Tests of AV43-6-G7 Potato and the Other 24 Plant Samples. **b** Amplification Plots and Standard Curve for the Event-specific Quantitative PCR Assay of AV43-6-G7 Potato. Amplification curves (the amount of the serially diluted genomic DNA of AV43-6-G7 potato was 100, 10, 1, 0.1, 0.01, and 0.001 ng AV43-6-G7 potato in a total of 100 ng DNA, respectively corresponding to 100 %, 10 %, 1 %, 0.1 %, 0.01 %, and 0.001 % of AV43-6-G7 potato haploid genome in a total of 100 ng of DNA per reaction) were generated to quantify AV43-6-G7 potato

Because there is little sequence-related information on the potato genome available from the GenBank database, it was impossible to determine whether the unknown DNA sequence (221 bp) was derived from the potato genome. Therefore, 1 pair of specific primers (Table 1) was designed based on the 470-bp DNA sequence, and qualitative PCR was performed using the DNA of AV43-6-G7 potato, GMO PH05-026-0048 potato, wild potato, and 24 other GMO plant lines as templates. As expected, specific DNA amplification was observed in AV43-6-G7 potato, GMO PH05-026-0048 potato, and wild potato, but unobservable in the other 24 GMO plant lines (Fig. 3). Hence, the unknown DNA sequence was derived from the potato genome.

### Specific for AV43-6-G7 potato of real-time PCR assay

Based on the revealed 5′-flanking sequence, event-specific primers were designed and used in a qualitative PCR assay of AV43-6-G7 potato. As expected, following the event-specific qualitative PCR assay of AV43-6-G7 potato, a passive real-time PCR amplification curve was obtained for AV43-6-G7 potato, whereas no amplification curve was observed for the other GM lines [26] (Fig. 3).

The amplification curves and standard curve were obtained for GM AV43-6-G7 potato using real-time PCR. (A) Specificity Tests of AV43-6-G7 Potato and Other 24 Plant Samples. (B) Amplification Plots and Standard Curve for the Event-specific Quantitative PCR Assay of AV43-6-G7 Potato. Amplification curves (the amount of the serially diluted genomic AV43-6-G7 potato DNA was 100, 10, 1, 0.1, 0.01, and 0.001 ng AV43-6-G7 potato DNA in a total 100 ng of DNA, respectively corresponding to 100 %, 10 %, 1 %, 0.1 %, 0.01 %, and 0.001 % of the AV43-6-G7 potato haploid genome in a total of 100 ng DNA per reaction) were generated to quantify AV43-6-G7 potato.

### Determination of the LOD of real-time PCR

To test the limit of detection (LOD) of the established method of event-specific real-time PCR, the DNA templates diluted along seven gradients were prepared at concentrations of 100 %, 10 %, 1 %, 0.1 %, 0.01 %, and 0.001 %, corresponding to 100, 10, 1,0.1, 0.01, and 0.001 ng AV43-6-G7 potato haploid genome in a total of 100 ng of DNA per reaction, respectively. A total of 4 parallel reactions were performed among triplicate samples. As expected, AV43-6-G7 potato DNA at 0.01 ng, corresponding to approximately 0.01 % ng of genomic DNA of AV43-6-G7 potato, was detected. The quantitative PCR results showed that the squared regression coefficient (R2) of the standard curve was 0.994 (Fig. 3). The high PCR efficiency and favourable linear relationship between the log DNA quantity and the fluorescence value (Ct) suggested that these primers and probes were suitable for quantitative measurements of AV43-6-G7 potato genomic DNA. Hence, the LOD of the event-specific quantitative PCR assay was 0.01 ng/action genomic DNA.

### Determining the LOQ of ddPCR

**Fig. 4:**
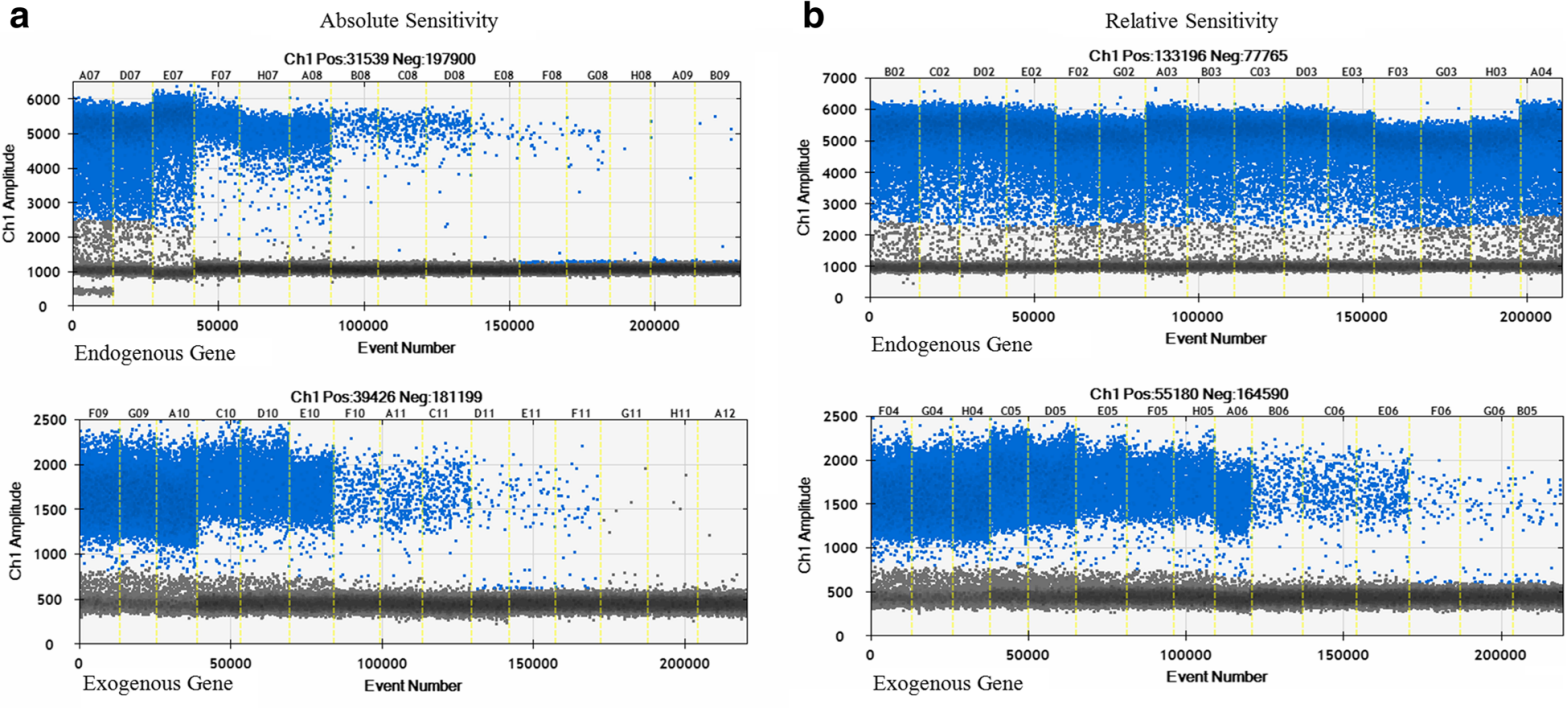
The digital PCR picture of serial concentration of genomic DNA in AV43-6-G7 potato in absolute sensitivity and relative sensitivity analysis. **a** Analysis of Absolute Sensitivity. DNA was diluted along five concentration gradients, including 10, 1, 0.1, 0.01, and 0.001 ng/μL, from left to right. For each concentration, three parallel samples were tested. **b** Analysis of Relative Sensitivity. Samples with a concentration of 100 %, 50 %, 10 %, 1 %, and 0.1 % of AV43-6-G7 potato haploid genome per reaction concentration are shown from left to right, respectively. Three parallel samples were tested for each concentration

Given the limitations of ddPCR for the quantification of GMO in food and feed samples, particularly at low target levels and in some complex matrices, generally accepted minimum performance requirements for analytical methods should be satisfied [27]. To avoid biases in comparing real-time PCR with ddPCR, inter-laboratory validation of real-time qPCR assays were alternatively used to validate the ddPCR using minimum adaptation. Therefore, apart from the master mix and settings specific to the QX100 droplet system, the primers and probe nucleotide sequences and concentrations, DNA concentration, and PCR thermo-profile were the same as those used in the real-time PCR assays (Fig. 4).

The absolute limit of quantification (LOQ) is the lowest target copy number in a sample that can be reliably quantified with an acceptable level of precision and accuracy [28]. In the absolute sensitivity analysis, all samples for determining dynamic range were prepared through serial dilution of a single stock AV43-6-G7 potato-specific DNA sample. The genomic DNA of AV43-6-G7 potato was tested along 5 concentration gradients. For each sample, copies of the endogene and flanking sequence were separately measured using ddPCR. For individual endogene targets and for the flanking sequence content, the coefficient of correlation R^2^ was 0.9849 and 0.9842, respectively (Additional file 2). The correlation coefficients obtained from ddPCR met the requirements (R^2^.0.98) set by the European Union Reference Laboratory for GM Food and Feed for acceptance of a quantitative PCR-based detection method for GMO [28] (Table 2).

**Table 2:** Relative sensitivity for the AV43-6-G7 potato

Prepared concentration	AV43-6-G7 potato genome DNA	Total template DNA	Copies of AV43-6-G7 potato genome	Group average concentration	Group RSD
100 %	50 ng/ reaction	50 ng/ reaction	57948.5	100.2 %	0.5 %
50 %	25 ng/ reaction	50 ng/ reaction	28974.5	53.4 %	0.2 %
10 %	5 ng/ reaction	50 ng/ reaction	5795.0	10.6 %	2.8 %
1 %	0.5 ng/ reaction	50 ng/ reaction	579.5	1.1 %	4.0 %
0.1 %	0.05 ng/ reaction	50 ng/ reaction	58.0	0.12 %	8.2 %

**Fig. 5:**
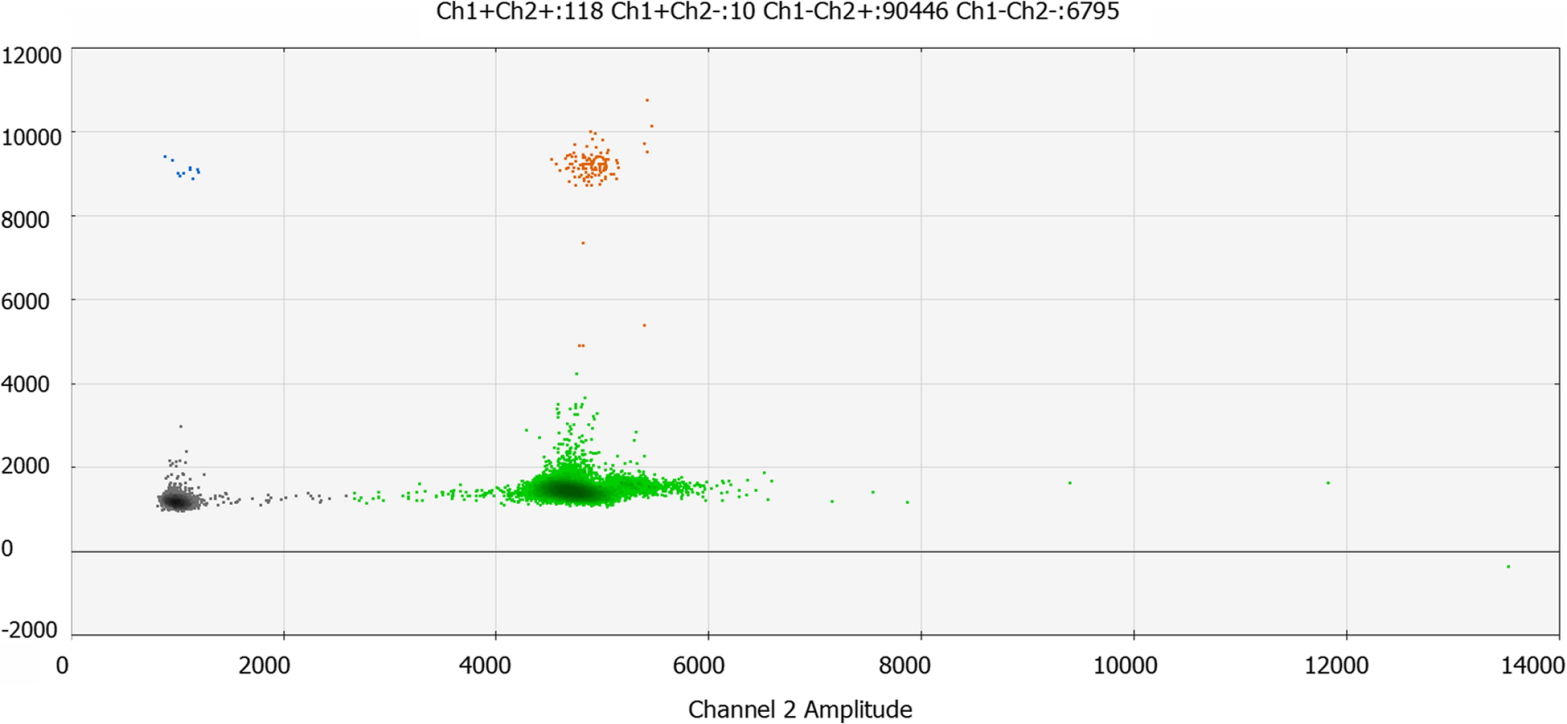
The amplification hot maps of ddPCR using the Bio-Rad system. For the different colours in C, black indicates negative for both FAM and VIC, the blue and green indicate positive only for FAM or VIC, and orange indicates positive for both FAM and VIC

In analysing relative sensitivity, the DNA template was diluted at a concentration of 100 %, 50 %, 10 %, 1 %, and 0.1 % of AV43-6-G7 potato haploid genome, respectively. When 0.1 % sample of AV43-6-G7 potato in a total of 50 ng/reaction potato DNA was detected, the experimental values and group RSD (relative standard deviation) were high, thus the accuracy of sample concentrations was higher than 0.1 % AV43-6-G7 potato in a total 50 ng/reaction potato DNA. The LOQ of each target group in the ddPCR assays was estimated as the lowest copy number within the dynamic with a relative standard deviation (RSD) of the measured copy number ≤25 % [28]. When the nucleic acid concentration is 50 ng per reaction, the 0.1 % concentration of the target nucleic acid can be quantified as 0.12 %, with an RSD of 8.2 %. The results in duplex droplet digital PCR reactions with 0.1 % target nucleic acid were quantified as 0.09 %, with an RSD of 9.7 % [13, 29] (Fig. 5).

This result was consistent with the requirements of the test methods [28]. Because the genome size of the potato is 840 Mb [30], the LOQ for ddPCR was 58 copies in a total of 50 ng DNA/reaction, consistent with a range from 30 to 100 copies in routine qPCR testing [22] (Table 3).

**Table 3:** Absolute sensitivity for AV43-6-G7 potato

DNA concentration of target	Copies of AV43-6-G7 potato genome	Group average	Group RSD
10 ng/reaction	11589.5	103.32 %	5.8 %
1 ng/ reaction	1159.0	107.31 %	3.5 %
0.1 ng/ reaction	115.9	93.39 %	8.7 %
0.01 ng/ reaction	11.6	97.29 %	17.6 %
0.001 ng/ reaction	1.2	95.14 %	24.9 %

## Discussion

This finding demonstrates that the AV43-6-G7 potato/hmg ddPCR assay can be performed over a wide range of target concentrations to determine the AV43-6-G7 potato content in a given sample and that values of approximately 11.6 copies constitute the range of quantification with ddPCR. The absolute limit of quantification (aLOQ) is the lowest target copy number in a sample that can be reliably quantified with acceptable precision and accuracy [31]. The aLOQ of ddPCR systems for hmg or AV43-6-G7 potato was estimated as the lowest copy number of 1.2 (Table 2), which was within the dynamic range with a coefficient of variation (CV) of the measured copy number of 25 % [28]. Based on this criterion, aLOQ was estimated at approximately 1.2 copies for the system. The results showed that ddPCR and real-time PCR have their own advantages. The applicability of ddPCR for GMO detection has previously been investigated and demonstrated as comparable to real-time PCR [32].

## Conclusions

In the present study, the 5′-exogene integration sequence was isolated, and event-specific qualitative and quantitative PCR assays were performed for identifying and quantifying Potato AV43-6-G7. The LOD of real-time PCR was 11.6 copies. A total of 58 copies, corresponding to a concentration of 0.1 %, reflected the relative sensitivity. In relative sensitivity experience, the lowest concentration was 0.1 %, which was considered practically sensitive, as this value was lower than the thresholds of 0.9 % in EU. The ddPCR assays of Potato AV43-6-G7 introduced and performed here achieved a limit of quantification of approximately 58 target copies with an RSD ≤ 25 %. The aLOQ of this system was approximately 1.2 copies. These results suggested that these methods are suitable for detecting the GMO content in internationally traded products.

## Additional files

Additional file 1: The names and sources of GM positive and negative samples used in the present study. (DOCX 14 kb)
Additional file 2: The copies of endogene and flanking sequence were separately measured using ddPCR in absolute sensitivity analysis. (XLSX 16 kb)

## Abbreviations

aLOQ: absolute limit of quantification
cdPCR: chamber digital PCR
CV: coefficient of variation
ddPCR: droplet digital PCR
dPCR: digital PCR
GMO: genetically modified organisms
LOD: limit of detection
LOQ: limit of quantification
NTC: non-template control
qPCR: quantitative PCR
R^2^: coefficient of correlation
RACE: rapid-amplification of cDNA ends
RSD: relative standard deviation
