# Supplementary material for: Event-specific detection of transgenic potato AV43-6-G7 using real-time and digital PCR methods
Source: BMC Biotechnol. 2016 Oct 27;16:74. doi: 10.1186/s12896-016-0303-8 (PMC5081928; doi:10.1186/s12896-016-0303-8)
Supplement: Additional file 1: — The names and sources of GM positive and negative samples used in the present study. (DOCX 14 kb) [file 12896_2016_303_MOESM1_ESM.docx]

Additional files 1 The names and sources of GM positive and negative samples used in this study

| Sample name | Source | Lot NO. |
| --- | --- | --- |
| GM EH92-527-1potato | AOCS* | AOCS 0806-C |
| GM AV43-6-G7potato | ERM# | ERM-BF431b |
| GM AM04-1020potato | ERM | ERM-BF430b |
| GM PH05-026-0048potato | ERM | ERM-BF435b |
| GM DAS-44406-6 soy bean | ERM | ERM-BF436b |
| GM DAS-68416-4 soy bean | ERM | ERM-BF432d |
| GM 305423 soy bean | ERM | ERM-BF426d |
| GM MON87701 soy bean | AOCS | AOCS 0809-B |
| GM MON87705 soy bean | AOCS | AOCS 0210-A |
| GM MON87708 soy bean | AOCS | AOCS 0311-A |
| GM BPS-CV127-9 soy bean | AOCS | AOCS 0911-C |
| GM GTS40-3-2 soy bean | ERM | ERM-BF410gk |
| GM MON89788 soy bean | AOCS | AOCS 0906-B |
| GM T304-40 cottonseed | ERM | ERM-BF429c |
| GM GHB119 cottonseed | ERM | ERM-BF428c |
| GM 281-24-236 x 3006-210-23cottonseed | ERM | ERM-BF422d |
| GM MON15985-7cottonseed | AOCS | AOCS 0804-D |
| GM MON863 maizes | ERM | ERM-BF416d |
| GM DAS-40278-9maize | ERM | ERM-BF433d |
| GM 98140maize | ERM | ERM-BF427d |
| GM MIR162maize | AOCS | AOCS1208-A |
| GM Bt11maize | ERM | ERM-BF412f |
| GM H7-1sugerbeet | ERM | ERM-BF419b |
| GM 73496rapeseed | ERM | ERM-BF434b |
| GM MON88913 Cotton seed | Shandong Entry-Exit Inspection and Quarantine Bureau | positive samples |
| rapeseed | Shandong Entry-Exit Inspection and Quarantine Bureau | positive samples |
| Corn liquor dregs（DDGS） | Shandong Entry-Exit Inspection and Quarantine Bureau | positive samples |

*：European reference materials，﹟：American Oil Chemists Society
